# Changes in different salivary biomarkers related to physiologic stress in elite handball players: the case of females

**DOI:** 10.1038/s41598-019-56090-x

**Published:** 2019-12-20

**Authors:** Gonzalo Mariscal, Pablo Vera, José Luis Platero, Fernando Bodí, Jose Enrique de la Rubia Ortí, Carlos Barrios

**Affiliations:** 1 Institute of Research on Musculoskeletal Disorders, Valencia Catholic University, Valencia, Spain; 2 Clínica Artes, Valencia, Spain; 3 Nursing School, Valencia Catholic University, Valencia, Spain; 4 Physics Faculty, University of Valencia, Valencia, Spain

**Keywords:** Applied immunology, Diagnostic markers

## Abstract

This study evaluates pre- and post-match concentrations of salivary cortisol, alpha-amylase (AA) and immunoglobulin A (lgA) in a group of 21 elite female handball players in the Spanish national women’s league. The players’ mean age was 23.0 ± 5.4 years. The concentration of the biomarkers was determined using ELISA. Mean playing time was 25.2 min. The players’ cortisol concentration increased significantly (*p* < 0.05) whereas the IgA concentration fell significantly (*p* < 0.01) at the end of the match. There were no significant changes in the concentrations of AA between pre and post-match timepoints. The changes observed in the study also depended on the position played; defenders exhibited the highest cortisol and lowest IgA concentrations after the match. Larger changes in cortisol and IgA were seen in those who played for more than 30 min. The present study shows that a competitive handball match increases physiologic stress in females, with activation of the hypothalamic-pituitary-adrenal axis and the adrenergic system, resulting in decreased immunocompetence.

## Introduction

Handball is a high-intensity game in which ~80% of each match is played with a workload intensity of over85% of the maximum heart rate^1,2^. Variables affecting this workload include the distance covered and speed, both of which are related to the position played. Defenders and forwards play at greater than 80% of maximum effective heart rate for a longer time than wingers^2^ and exhibit higher mean and maximum heart rates. For years, the quantification of certain salivary markers has been used to establish a player’s physiologic and immune endocrine state, specifically their response to exercise^3^. In fact, given its non-invasive nature, it is now more common to analyse salivary biomarkers in both athletes and non-athletes^4–6^.

One of these markers is cortisol, a steroid hormone that is a member of the glucocorticoid family. It is secreted from the suprarenal cortex through the hypothalamic-pituitary-adrenal (HPA) axis and increases in response to stressful factors including physical effort^7,8^, rising proportionally with duration and intensity. An “intensity threshold” has been proposed at ≥60% VO2 max in exercise lasting 20–30 minutes, beyond which a significant increase in serum cortisol is seen^9,10^. The concentration of salivary cortisol serves as a proxy for that of serum cortisol, whether at rest or exercising^11,12^. Significant correlations have been described between salivary and serum cortisol concentrations after intense exercise, a 30 second Wingate test^13–15^, and some sports competitions^16–18^. Salivary cortisol reflects the biologically active fraction of the total serum cortisol and some studies have shown that the change in cortisol in response to exercise is sharper in saliva than in blood^14,19^, making salivary cortisol more sensitive and giving a more accurate measurement of the dynamic activity of the HPA axis. In addition, monitoring cortisol in athletes may more accurately reflect the response to training^5,13,16^.

Alpha-amylase (AA) has been described as a physiologic stress biomarker that reflects the activity of the sympathetic nervous system^20^, especially during physical activity^21^. In fact, high concentrations of AA, epinephrine, and norepinephrine have been described in saliva after aerobic activity^22^. These data back the use of salivary AA as a proxy for the increase in catecholamines induced by exercise^23^. Specifically, the determination of salivary AA concentration by immunologic methods such as ELISA has been directly related to adrenergic activity^24^. Based on the existing evidence regarding exercise-induced salivary secretion of cortisol and AA, it has been proposed that the identification of these substances could be used to establish a more physiologic prescription for training schedules and close monitoring of athletes’ recuperation^3^.

Physiologic stress also affects the immune system. Immunoglobulin A (IgA) is an important aspect of this system that is particularly present on mucous membranes. It can be used to determine immune capacity as well as physiologic well-being following some types of physical therapy^25–28^. An immediate drop in salivary IgA has been described after prolonged exercise, generally recovering within 24 hours^20^. The intensity of the exercise may also have an influence on the salivary IgA; a rise in salivary IgA has been described following highly intense exercise at >80% VO2 max for less than 2h^20,29^. As a consequence, determining the IgA concentration in saliva may be useful in identifying an excessive training workload (phase of overtraining). Furthermore, it could also determine the risk of respiratory infection in professional athletes; it has been shown that a sustained drop in IgA secretion is associated with an increase in salivary cortisol levels, which demonstrates that high levels of cortisol may act as a precursor to the suppression of the mucosae membrane’s immunity^30^. This may help explain the higher prevalence of pneumonia, bronchitis, bronchopneumonitis and the common cold in professional athletes^31^. All these evidences justify the importance of monitoring and controlling the changes in the secretion of these three molecules in elite sport, and specifically in handball. This sport modality is characterized by intense and repeated efforts in short periods of time throughout a match. The quantification of cortisol, AA and IgA could illustrate the amount of physiological stress and the evaluation of a correct recovery. The monitoring of these molecules could help to prevent overtraining and other physical problems that can influence sports performance.

The aim of this study was to evaluate the dynamics in the secretion of cortisol, AA, and IgA in saliva before and after a match in a group of professional handball players, taking into account factors such as their position and playing time. Our hypothesis is that quantifying the secretion of these molecules could be used to determine the physiologic response to exercise in professional sports.

## Results

### Anthropometric profile

The anthropometric characteristics of the 21 handball players included in the study are shown in Table 1. There were no statistically significant differences between the two groups (wingers/forwards and defenders) in any of the variables analysed.

**Table 1:** Age, anthropometric data (n = 21); SD: Standard Deviation.

	Whole sample	Wingers & Forwards (n:10)	Defenders (n:11)	Mann-Whitney test	
	Mean ± SD	Mean ± SD	Mean ± SD	Z	p
Age (years)	23.0 ± 5.4	25.0 ± 6.3	21.0 ± 3.2	−1.487	0.137
Weight (kg)	67.1 ± 7.5	64.9 ± 5.9	69.2 ± 8.6	−0.420	0.674
Height (cm)	170.2 ± 4.1	168.7 ± 3.2	171.6 ± 4.6	−0.583	0.560
BMI (kg/m^2^)	23.1 ± 2.0	22.8 ± 2.2	23.4 ± 1.9	−0.053	0.958
Fat (%)	28.9 ± 5.7	28.6 ± 3.6	29.3 ± 7.5	−0.525	0.599
Muscle (%)	31.1 ± 2.7	31.1 ± 1.3	31.1 ± 3.7	−0.315	0.753
Basal metabolism (calories)	1340.5 ± 328.3	1227.1 ± 438.6	1453.9 ± 96.0	−1.524	0.130

### Variation of biomarkers during the match

**Figure 1:**
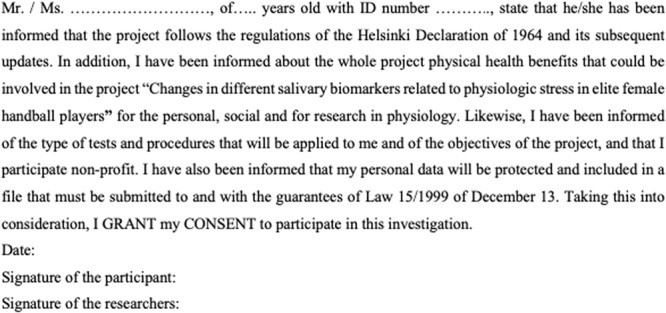
Salivary concentration of biomarkers before and after the match. (**a**) Cortisol. (**b**) Alpha-amylase. (**c**) IgA. Wilcoxon rank test was used for differences between pre and post-match determinations. Statistically significant differences between groups are reported (P < 0.01). Data are shown as Tukey’s box plots. Cortisol values increased significantly after the match (**a**). As compared to values before match, no significant changes were recorded in the concentrations of AA after the match, although median increased slightly (**b**). IgA concentration fell significantly in the samples taken at the end of the match (**c**).

Cortisol values showed a statistically significant increase after the match (p < 0.01) with a mean difference of 4.77 ng/ml (95% CI 1.74–7.81), reflecting a 1.1-fold increase. Conversely, there was a statistically significant fell in IgA concentrations after match (p < 0.01) with a mean difference of 495.5 µg/ml (95% CI 223.48–767.93), reflecting a 0.6-fold decrease. The variation in the concentration of AA was no statistically significant (p = 0.289) with a slight mean increase of 113.7 U/ml (95% CI 109.02–336.53), a small 0.12-fold increase. Figure 1 shows the concentrations of salivary cortisol, AA, and IgA among all players before and after the match.

### Variation of biomarkers in relation to the position of the players into the field

**Figure 2:**
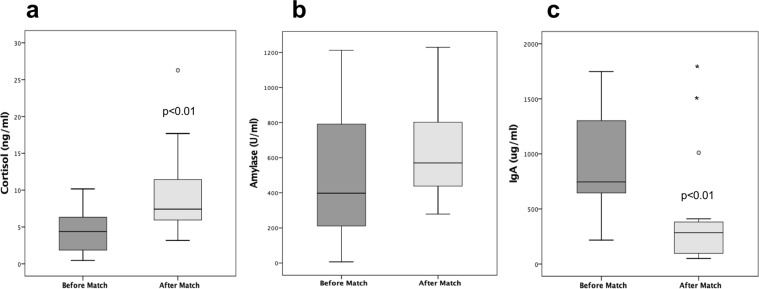
Mean fold change in concentration of salivary biomarkers after the match according to the players’ position. Negative values indicate decrease in concentration. Wilcoxon rank test was used for differences between pre and post-match determinations. Statistically significant differences between groups are reported (*P < 0.05; **P < 0.01). At the end of the match, only defenders (n = 8) showed a significant increase in cortisol (3.19 folds). Both groups of players showed a significant decrease in salivary IgA concentration at the end of the match (−0.75 in wingers and forwards; −0.51 in defenders). Defenders showed a slight increase in AA as compared to wingers and forwards (n = 11), but the increase was not statistically significant.

Defenders/goalkeepers showed statistically significant changes (p = 0.036) in cortisol not seen in wingers/forwards (p = 0.091). The average increase in cortisol was greater in defenders/goalkeepers than in the wingers/forwards (8.08 ng/ml, 95% CI 2.31–13.84 vs 1.77, 95% CI −0.29–3.84; p = 0.024). The defenders/goalkeepers also experienced a greater but non-significant increase in AA. The drop in IgA was significant among all players, but greater in the wingers/forwards (p = 0.003) than in the defenders/goalkeepers (p = 0.036). However, differences in the IgA concentration was not statistically significant in any of the two group of players. Figure 2 summarizes the fold change in the biomarkers according to player position. Defenders showed the highest increase in cortisol. Both groups of players showed an almost similar decrease in salivary IgA concentration.

### Influence of play time on the variation of biomarkers

**Figure 3:**
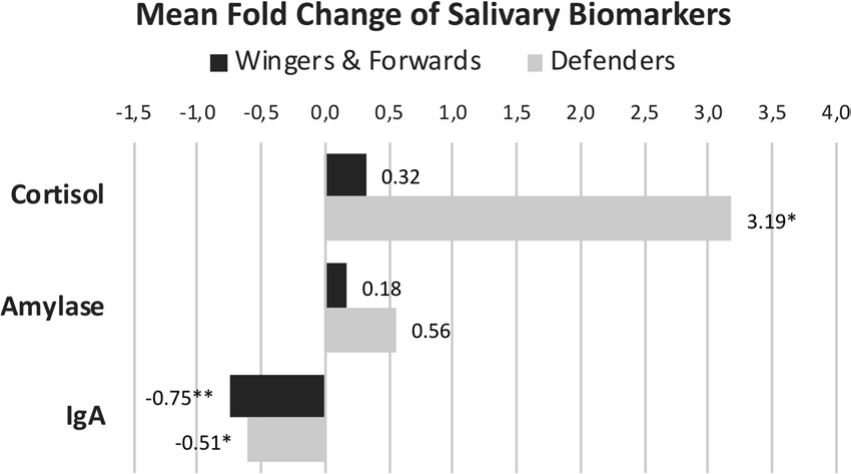
Salivary concentration of biomarkers before and after the match according to the playing time (less or more than 30 minutes). (**a**) Cortisol. (**b**) Alpha-amylase. (**c**) IgA. Wilcoxon rank test was used for differences between pre and post-match determinations. Statistically significant differences between groups are reported (P < 0.05). Data are shown as Tukey’s box plots. The group of participants playing more than 30 minutes (n = 7) showed a significant increase in cortisol. Participants playing less than 30 minutes (n = 14) showed a significantly higher decrease in salivary IgA than players been involved in the match for more than 30 min.

The mean playing time was 25.2 min. (95% CI 17.8–32.5), which was 42% of the match’s duration. Wingers and forwards played an average of 23.8 min. (95% CI 10.2–37.5), and defenders 26.5 min. (95% CI 16.8–36.1). A non-significant difference was found between the groups (p = 0.563). When players were divided according to the duration of play (at least half vs less than half the match), differences in the concentrations of the biomarkers were appreciated (Fig. 3). Both groups showed an increase in cortisol that was only statistically significant in the former (5.01 ng/ml, 95% CI 0.30–9.71; p = 0.018 vs 4.48, 95% CI 1.95–10.91; p = 0.110). Interestingly, the latter group showed a higher decrease in salivary IgA (545.11 ng/ml, 95% CI 127,45–962,77; p = 0.015 vs 269.81, 95% CI −392.66–932.30; p = 0.310) and almost no change in AA (15.63; 95% CI −296.26–327.53; p = 0.953). The group that played for more than 30 minutes showed a higher increase in salivary AA, which was also not statistically significant (148,99; 95% CI −645.39–347.40; p = 0.398) (Fig. 3).

Among the whole sample, there was no correlation between the time played and increases in cortisol (Spearman’s rho = 0.147; p = 0.586) and AA (Spearman’s rho = 0.479; p = 0.061), or with the drop in IgA (Spearman’s rho = −0.038; p = 0.888).

## Discussion

High physiologic stress is expected in any athlete after a highly competitive match. It is critical for those responsible for the conditioning and health of elite athletes to optimize recovery from this stress as such is crucial for protecting athletes’ health and to improving their performance. It thus becomes necessary to monitor the concentrations and variations of stress biomarkers after competition, allowing one to establish the demands for subsequent recovery; avoiding even greater competitive stress, overtraining, fatigue, sports injuries, related diseases, and exhaustion^32,33^. Controlling all these factors has been related to an improvement in performance^34^.

The activity of the HPA axis and the sympathetic adrenomedullary system, on which cortisol and AA secretion respectively depend^35^, play a fundamental role in physiologic stress. The salivary samples of female handball players after a highly competitive match showed an increase in cortisol, and to a lesser extent in alpha amylase. The increase in salivary cortisol found in this study is in concordance with the increase described after a rugby match^36^, a kick boxing fight^37^, a taekwondo fight^38^and a Bruce test on a treadmill in physically active males^39^, all of which – like handball – require intense, sustained effort. The demands of professional competition also seem to have an influence; a decrease in cortisol secretion is seen in training among teenagers in school^40^.

We did not find a significant increase in AA, even though the response of AA to exercise has been described as similar to that of cortisol^41,42^. In addition, salivary AA is a more sensitive biomarker than cortisol, since it is produced in the salivary glands instead of being transported from blood to saliva^29^. In fact, Kivlighan and Granger (2006) found higher levels of salivary AA than of cortisol in response to an extreme test on a stationary bicycle^21^. A high concentration of salivary AA was also found in young athletes immediately after a taekwondo championship^43^. These sports require bursts of intense effort; the disagreement with our results may be due to the intensity and duration these players underwent; long, low-moderate intensity exercise does not affect the rate of salivary AA secretion^43^. As the players in our study underwent a sustained effort, this could explain our results of a non-significant increase in AA.

The responses of salivary IgA to high-intensity exercise are not consistent among different studies. Some detected a drop in IgA after effort^44–46^, others detected a rise^47,48^, and yet others detected no change^49,50^. There are very few studies that examined the response of salivary IgA after a sports competition and when they do, they do not normally show the change over the match^51,52^. This drop we found in IgA could be linked to the duration and activity involved in handball, similar to that of cycling^20^, kayaking^46^, rowing^47^ and marathons^48^, all of which require a prolonged effort and resulted in a drop in IgA. This drop could be an indirect marker of fatigue as physiologic stress could be related to a loss of physiologic well-being, which IgA is considered to be a marker of^53^. It should be noted that our results have been compared, in all cases, with those obtained in studies conducted in male athletes. This aspect should be taken into account when drawing conclusions. However, the secretion pattern for each molecule analyzed seems to be similar between men and women. Specifically, after intense physical exercise no differences in salivary secretion of cortisol^54^ or IgA^55^ were observed. Furthermore, after a stressful stimulus the salivary secretion of AA was also similar in both genders^56^.

We found that the position played had an impact on the players’ physiologic stress, with the defenders showing a greater rise in cortisol. As these results are in line with those obtained in other studies, it seems that defenders make a greater physical effort than players in attacking positions, specifically wingers^2^. The wingers/forwards had the greatest decrease in IgA concentrations, which could indicate a greater sustained intensity apparently not associated with greater physiologic stress.

No direct relationship between the time played (as a binary variable) and the change in the biomarkers was found. However, those who played for more than 30 min showed a statistically significant rise in cortisol and drop in IgA. This suggests that the activation or deactivation of some of these biomarkers is dependent on thresholds of cumulative activity. The relevance for trainers and coaches is that rotating players should be considered to avoid prolonged periods of playing time.

The results of this study open up the possibility of using these markers to determine the physiologic stress athletes undergo in different sports and which positions make the greatest exertion. Certainly, the scenario of the current study shows a relevant application for the use of salivary biomarkers in sports sciences. Our study’s most important limitation was that it included only women. In this study, the phase of the menstrual cycle could not be controlled; not attempting to do so has the advantage of reflecting the reality of sports competitions.

Another limitation was the complexity of controlling the players’ hydration, which could have influenced the biomarker concentrations. We also could not control nutritional status despite the recommendations to the players to avoid the intake of some types of energy foods that could influence the secretion of cortisol or AA. Furthermore, the secretion of these biomarkers might be determined after different periods in a match so as to explore their secretion dynamics and thus at the physiologic stress the players undergo throughout the match.

In conclusion, after a highly competitive match of female handball, salivary cortisol and AA concentrations increased while IgA concentrations decreased. In addition, the position of the players was shown to impact the secretion of these biomarkers, with defenders exhibiting the highest cortisol and lowest IgA post-match concentrations. It also seems players should surpass a certain amount of play time to show significant changes in the concentration of these biomarkers. All these results suggest that the HPA axis, the adrenergic, and the immunological systems are altered by moderate-intensity exercise that produces an increase in physiologic stress. However, these finding should not be generalized to male handball players or other sports with similar intensity requirements. Further studies are needed to deeply understand the role of cortisol, AA, and IgA as markers of physiologic stress in competitive sports.

## Methods

### Structure of the study

The saliva samples were collected 5 minutes before and 10 minutes after the last official match in the Spanish national women’s league. The match had two halves of 30 minutes with a break of 15 minutes. To homogenize sample collection, all players were recommended to hydrate just before the start of the game with 250 ml of water, and to do so again at rest and as needed. It was emphasized that hydration should be only with water and that they do not ingest any food, following the recommendations of other authors^57^.

### Participants

Saliva samples were requested from the 32 players who made up the two teams. However, we only analysed samples from 21 players (5 were from the visiting team). These participants were assigned to two groups: wingers/forwards (n = 10), and defenders (n = 11). Anthropometric data are shown in Table 1. Neither the stage of the players’ menstrual cycle nor the use of oral contraceptives was evaluated. Prior to the study, all of the players had been training daily for 120–150 minutes per session, 5 days a week. They had also played one official match each week.

### Saliva collection and analysis

Players were asked to wash their mouths out with distilled water to avoid altering the samples with traces of food containing acid or sugar. At least 3 ml of saliva was passively collected (gold standard drool method) in a 10 ml plastic sterilised tube and then placed in an ice container. The supplies were centrifuged in the laboratory at 1,500 G for 15 minutes and the supernatant was stored frozen in micro-tubes at −20 °C until the samples were analysed^16^. Once defrosted to room temperature, the cortisol, AA, and IgA concentrations of each saliva sample were determinedvia (ELISA) following standard protocols and control values given by the provider (DRG International Inc). The ELISA kits used in the study were: (i) SLV-2930 for salivary cortisol; (ii) SLV-4636for salivary IgA, and (iii) EIA-5836for salivary amylase.

### Recording the playing time

The playing times of each of the players was recorded by analysing a video of the match. Video recordings included a digital timer. Each player was followed individually along the match and the total playing time period was a sum of the different periods played.

### Statistical analysis

The anthropometric variables of the total sample are presented as mean and standard deviations.95% confidence intervals were also calculated. To determine the distribution of the data, the Shapiro-Wilk test was used before choosing the most appropriate statistical model. The Shapiro-Wilk test found that the pre-match AA and the post-match cortisol and IgA were not normally distributed. Thus, the concentration of salivary biomarkers was given in median and interquartile ranges (IQR). Furthermore, given the size of the sample, non-parametric tests were used for the statistical analysis. In order to compare the cortisol, AA, and IgA concentrations before and after the match, the Wilcoxon test for related variables was carried out. A Mann-Whitney test was used to analyse differences in biomarkers between wingers/forwards and defenders/goalkeepers as well as between players based on whether they played for at least half the match. To evaluate the relationship between the time played and the post-match biomarker concentrations as well as the change in the concentrations from the start to the end of the match, the non-parametric Spearman’s correlation coefficient (rho) was analysed. All of the statistical analyses were carried out with the SPSS 24 pack for Mac (SPSS Inc., IBM Company, USA). The level of significance was set at p < 0.05.

### Informed consent

**Figure 4:**
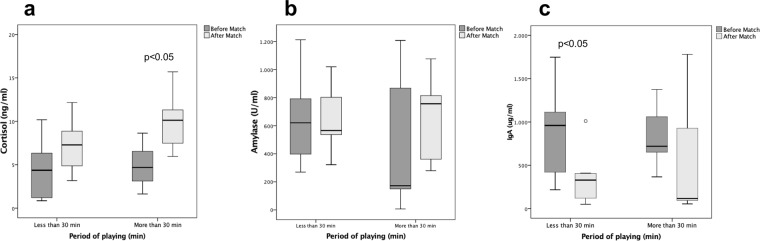
Informed consent statement for study participation.

All patients were informed of the study and signed the following document agreeing to participate in it (Fig. 4):

Mr./Ms. ………………………, of…. years old with ID number ……….., state that he/she has been informed that the project follows the regulations of the Helsinki Declaration of 1964 and its subsequent updates. In addition, I have been informed about the whole project physical health benefits that could be involved in the project “Changes in different salivary biomarkers related to physiologic stress in elite female handball players**”** for the personal, social and for research in physiology. Likewise, I have been informed of the type of tests and procedures that will be applied to me and of the objectives of the project, and that I participate non-profit. I have also been informed that my personal data will be protected and included in a file that must be submitted to and with the guarantees of Law 15/1999 of December 13. Taking this into consideration, I GRANT my CONSENT to participate in this investigation.

Date:

Signature of the participant:

Signature of the researchers:

### Ethical approval

All the participants provided their consent to take part in the study. The study was approved by the ethical committee of the University Catholic of Valencia (UCV/2015–2016/62). The entire study adheres to the Declaration of Helsinki for research involving human subjects.

## Data Availability

The datasets generated during and/or analyzed during the current study are available from the corresponding author on reasonable request.
